# Asymptomatic uterine metastasis of breast cancer: Case report and literature review

**DOI:** 10.1097/MD.0000000000031061

**Published:** 2022-10-14

**Authors:** Dechen Kong, Xiaotong Dong, Peiyan Qin, Daqing Sun, Zhengtao Zhang, Yan Zhang, Furong Hao, Mingchen Wang

**Affiliations:** a Clinical School, Weifang Medical University, Weifang, China; b Department of Pathology, Weifang People’s Hospital, Weifang, China; c Department of Radiation Oncology, Weifang People’s Hospital, Weifang, China; d Weifang Key Laboratory of Radiophysics and Oncological Radiobiology, Weifang, China.

**Keywords:** asymptomatic metastasis, breast cancer, case report, diagnosis, GATA-3, uterus metastasis

## Abstract

Uterine metastasis from breast cancer is extremely rare. Asymptomatic patients with cervical metastases from breast cancer are rarer and more likely to be missed. We present an asymptomatic patient with breast cancer metastasized to the uterus and share opinions on diagnosing and treating for this kind of cases.

**Patient concerns:**

We present the case of a 64-year-old woman who was diagnosed with both breast cancer and uterine fibroids after examination. She had no symptoms of gynecological disease during breast cancer treatment. A positron emission tomography/computed tomography (PET/CT) scan was performed during reexamination, revealing multiple metastases of the bone throughout the body and an abnormal hypermetabolic mass in the uterus. It was later confirmed as uterine metastasis by pathology.

**Diagnosis:**

A diagnosis of metastatic breast invasive lobular carcinoma was established after a uterine curettage.

**Interventions and outcomes:**

Treatment of the uterine metastasis included systemic chemotherapy, total abdominal hysterectomy and bilateral salpingo-oophorectomy (TAH and BSO), postoperative radiotherapy, and postoperative chemotherapy. The patient eventually refused further treatment for personal reasons and died at home.

**Lessons:**

Breast cancer metastases to the uterus are very rare and further research is needed for their diagnosis and treatment. During reexamination of breast cancer patients, clinicians must be alert to metastasis to gynecologic organs. This is particularly important in hormone receptor-positive patients with asymptomatic distant metastasis.

## 1. Introduction

Breast cancer is the most common tumor in women and the main cause of cancer-related death among women worldwide. In 2020, there were approximately 2.3 million new cases of breast cancer globally, with more than half being diagnosed in low-income and middle-income countries, and 685,000 people died because of breast cancer.^[1]^ Terminal breast cancer can metastasize to many organs, mostly the bone, liver, lung, and brain, though genital metastasis of breast cancer is rare. A large number of investigations and many examples of female reproductive system metastasis have proven that the ovary and vagina are the main metastatic sites of secondary tumors, but metastasis to the uterus is not common.^[2]^ Here, we report a patient with breast cancer and asymptomatic uterine metastasis; we discuss and review the clinical diagnosis, treatment course and pathological features.

## 2. Case presentation

**Figure 1.**
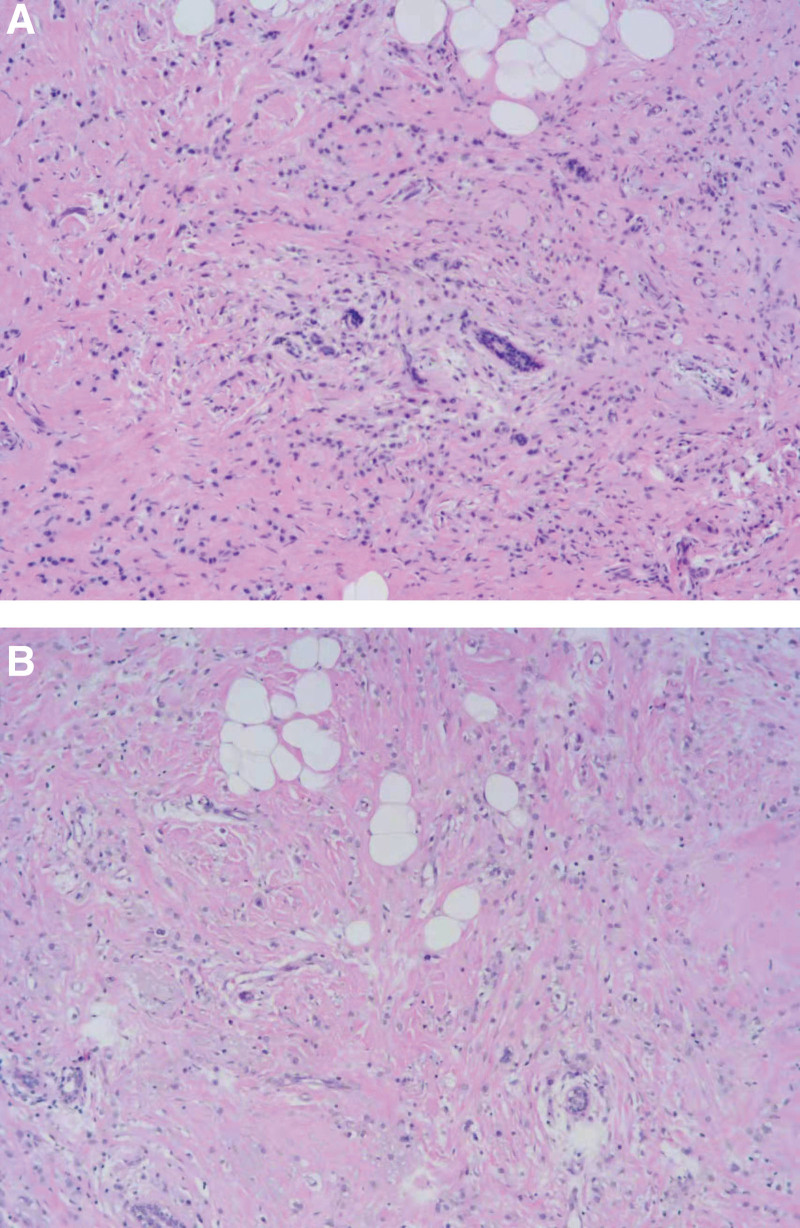
A, B: Pathological photographs after modified radical mastectomy. (hematoxylin and eosin, original magnification × 100).

A 64-year-old woman from Shandong Province, China, was admitted to our hospital on April 12, 2021; she had a history of breast cancer operated on 4 years prior, with multiple metastases. The patient visited another hospital in July 2017 for a tumor in the right supraclavicular region, and a right breast tumor was found. Before the operation, only contrast-enhanced computed tomography (CT) and color doppler ultrasound were used to examine the uterus, suggesting uterine fibroids, and no biopsy was performed because there were no obvious clinical symptoms. On July 14th, 2017, modified radical mastectomy for right breast cancer was performed in another hospital. The postoperative pathological finding was infiltrating lobular carcinoma (ILC) of the breast. The volume of the tumor was 1.5 cm × 1 cm, and the regional lymph node status was as follows: L1 group (27/30), L2 group (3/3), and L3 group (5/5). The immunohistochemistry prompted P120(enchylema+), 34βE12(+), E-cadherin(-), ER(strongly positive + 70%), PR(-), HER-2(-), Ki-67(<5%), P53(-), EGFR(-), PD-L1(-), and PD-1(-). Accordingly, the patient was diagnosed with infiltrating lobular carcinoma of the breast (pT1cN3cMx, stage IIIC by the American Joint Committee on Cancer, 8th edition) (Fig. 1). After surgery, she received 4 cycles of chemotherapy with epirubicin (100 mg/m² on Day 1) and cyclophosphamide (600 mg/m² on Day 1), and then paclitaxel was used for 4 cycles (specific dose unknown). Intensity-modulated radiotherapy of the right chest wall and the region of the supraclavicular lymph nodes was initiated, with a total dose of 50 Gy in 25 fractions. However, the patient refused treatment after 24 rounds of radiotherapy and was discharged from the hospital. She was given endocrine therapy with letrozole.

**Figure 2.**
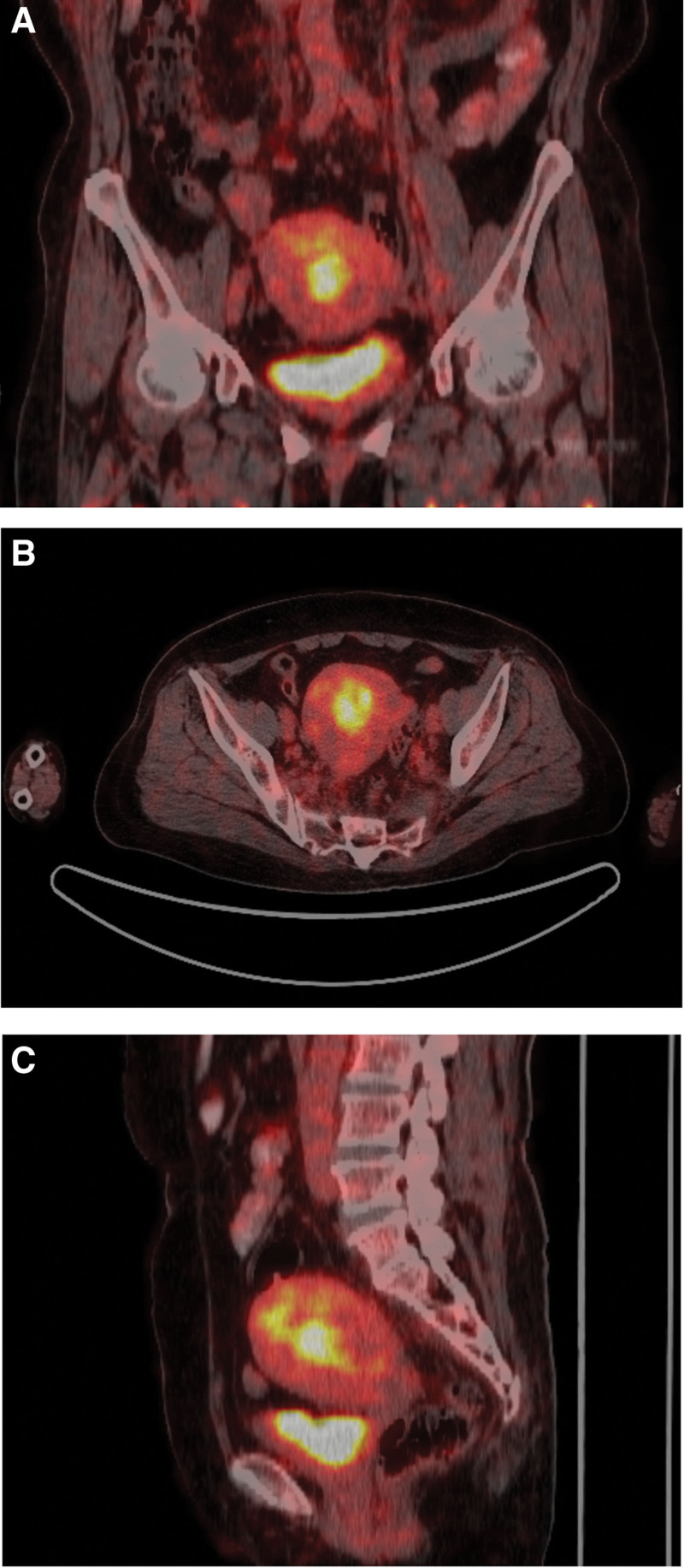
Abnormal high metabolic signal of the uterus by PET-CT examination. (A: PET/CT pelvis – axial view; B: PET/CT pelvis – coronal view; C: PET/CT abdomen & pelvis – sagittal view).

**Figure 3.**
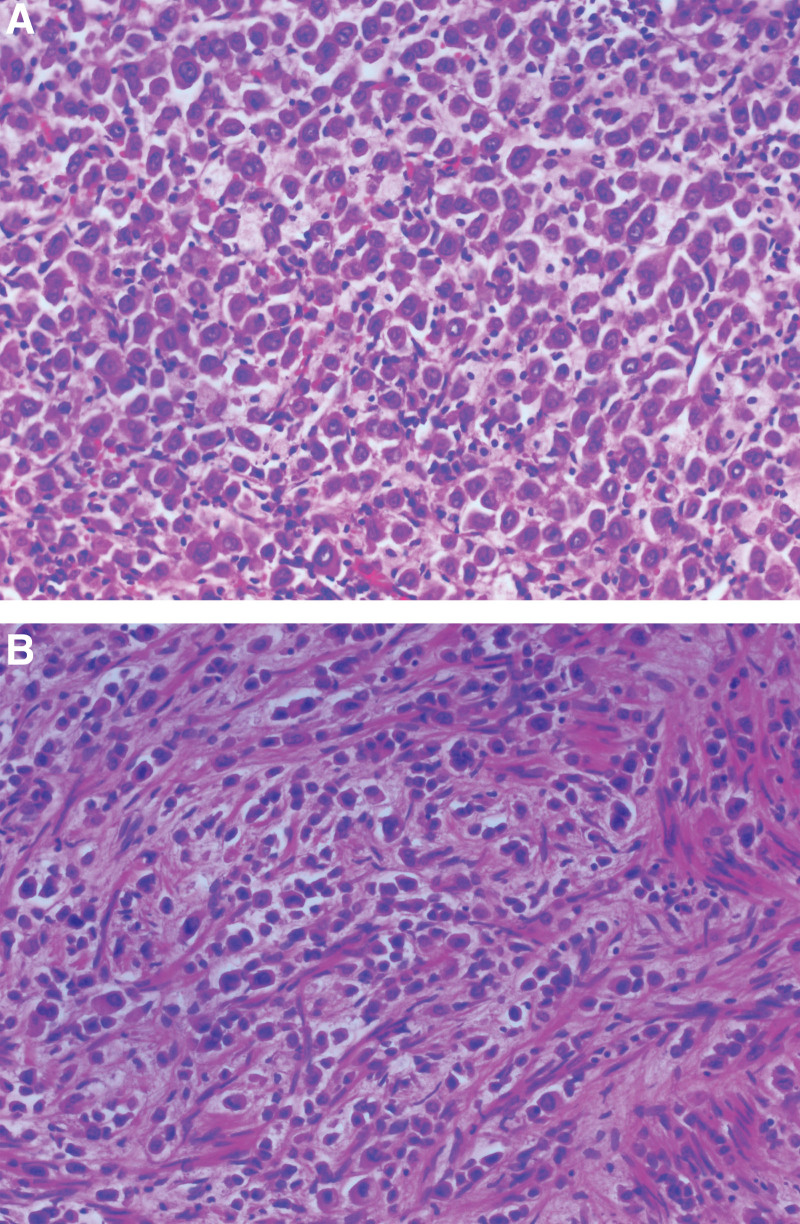
Pathological photographs after total abdominal hysterectomy and bilateral salpingo-oophorectomy (TAH and BSO). A: Metastasis of breast cancer to the uterine wall. B: Metastasis of breast cancer to uterine leiomyoma. (A-B, Hematoxylin and eosin, original magnification × 200).

The patient developed intermittent pain in the right shoulder in December 2019. The results of positron emission tomography/computed tomography (PET/CT) examination indicated multiple bone metastases in the left acetabulum, the left femoral head, the left ischium and the left transverse process of T4, T7, and L5. There were multiple enlarged lymph nodes in the left neck and left clavicle, and metabolism was slightly higher. As the uterine body showed abnormally high metabolism, metastasis was considered (Fig. 2). We began radiotherapy and simultaneous zoledronate therapy for the bone metastases on December 4, 2019. Uterine curettage was performed on December 6th, 2019, and pathological biopsies were taken. Combined with medical history and immunohistochemistry, the results showed malignant tumors consistent with invasive lobular carcinoma of metastatic breast origin. Immunohistochemistry revealed GATA binding protein 3 (GATA-3) (+), P120(enchylema +), E-cadherin(-), ER(3+, 2%), PR(2+, 2%), C-erbB-2(1+), P53(1+, 2%), Vimentin(-), CD10(-), and Ki-67 (35%). The patient has been taking oral endocrine therapy after surgery, but the effect is not good. And the patient refuses to take second-line endocrine therapy for economic reasons. Considering that the patient has advanced breast cancer with multiple metastases throughout the body, she received paclitaxel liposome (175 mg/m² on Day 1) + capecitabine (1000 mg/m² on Days 1–14) chemotherapy for 6 cycles and was admitted for total abdominal hysterectomy and bilateral salpingo-oophorectomy (TAH and BSO). Combined with her history and immunohistochemistry, postoperative pathology suggested invasive lobular carcinoma originating from breast cancer, with an area of 1.5 cm × 1 cm and a thickness of 2.5 cm. All layers of the uterine wall were involved. The nerves, cervix, and double appendages were also invaded, and we found tumor thrombi in the vessels. We also observed cancerous cells inside the uterine leiomyoma, with a volume of 2 cm × 2 cm × 1.7 cm; immunohistochemistry was ER(-), PR(1+,1%), C-erbB-2(0), GATA-3(+), P120(enchylema +), E-cadherin(-), CK7(+), CK20(-), CDX2(-), SATB-2(-), and Ki-67(5%) (Fig. 3). Postoperative radiotherapy was directed at the surgical cavity and the region of the pelvic lymph nodes, and chemotherapy of capecitabine (1000 mg/m² on Days 1–14) was administered simultaneously. After that, zoledronate therapy was administered in our department every month to inhibit bone metastasis. On February 25th, 2021, imaging reexamination showed that the left supraclavicular lymph node had increased in size, and metastasis of breast cancer was confirmed by pathology. Then, the patient was given gemcitabine (1000 mg/m² on Days 1 and 8) + cisplatin (75 mg/m² on Days 1–3) for 2 cycles of chemotherapy. However, the patient withdrew from further treatment for personal reasons and died in August 2021.

PET/CT = positron emission tomography/computed tomography.

## 3. Discussion

Cancer is not only the main cause of death worldwide but is also the leading cause of death in women. According to the latest research in 2020, breast cancer has become the cancer with the highest incidence in women. Among new cancer patients in 2020, the morbidity of breast cancer (11.7%) surpasses that of lung cancer (11.4%).^[1]^

The most common metastatic site of breast cancer is the lung, followed by the bone, liver, brain, and soft tissue; in contrast, metastasis of the uterus is not common. The metastatic mechanism of breast cancer may be related to loss of E-cadherin protein expression on the tumor cell membrane, a significant pathological feature of ILCs. This may be due to inactivation of the CDH1 gene at 16q22 by a variety of mechanisms, including CDH1 gene mutation, chromosome loss and promoter methylation.^[3]^ Lack of the E-cadherin leads to dysfunction of the E-cadherin-catenin complex, which affects adhesion between cells.^[4]^ It may explain the specific metastatic pattern of ILCs. Invasive lobular carcinoma has been indicated as the most common pathological type of metastatic breast cancer. More than 70% of pathological types are invasive ductal carcinoma, and invasive lobular carcinoma accounts for only 5% to 15%.^[5]^ The common sites of invasive ductal carcinoma (IDC) metastasis are the lung, bone, and liver. Metastatic dissemination of ILC is more common than of IDC to the bone and other organs (including the peritoneum, ovary, gastrointestinal tract, skin, adrenal gland, gallbladder, pancreas, kidney, bladder, eyelid).^[6]^ Clinical autopsy comparison of IDC and ILC shows that ILC spreads more easily to gynecological organs than does IDC.^[7]^

The female reproductive system is rarely affected by metastasis. The most common site of metastasis is the ovary, followed by the vagina (75.8% and 13.4%). With no relationship to the location of the primary tumor, only 8.1% of cancers metastasize to the uterus.^[2]^ The ovary is very vascular, with a well-developed lymph network, with inherent pH, partial pressure of oxygen and metabolic factor characteristics. It is easy for tumor cells to colonize this tissue.^[8]^ Conversely, the uterus contains more fibrous tissue, which is not conducive to the dissemination and implantation of tumor cells.

Uterine metastasis from breast cancer is extremely rare. Patients with asymptomatic uterine metastasis are even rarer. Here we summarized asymptomatic patients of breast cancer with metastases to the uterus (Table 1). Most patients with asymptomatic metastases were regularly followed up because they were hormone receptor positive and took tamoxifen. Tamoxifen use increases the risk of endometrial carcinoma due to its partially agonistic effect on endometrium^[18,19]^ and most commonly presents with vaginal bleeding.^[20]^ Other asymptomatic patients were initially diagnosed with an abnormally enlarged uterus, which was later confirmed to have metastasized breast cancer. The diagnosis of these patients with asymptomatic metastases is incidental and more likely to be missed than patients with abnormal vaginal bleeding symptoms. Some studies have shown that among common cancers, breast carcinoma, and ovarian carcinoma have higher asymptomatic rates.^[21]^ This creates barriers for doctors to diagnose patients with advanced breast cancer. In this study, contrast-enhanced CT and color doppler ultrasound showed that the patient has uterine fibroids at the initial diagnosis. But she had no previous symptoms of gynecological disease, nor any history of gynecological disease, and therefore no further diagnosis was made. Whether the patient has distant metastases at the outset is also unknown. Is there a risk of distant metastasis of uterine myoma? It’s very important to ensure timeliness of detection and diagnosis in patients with breast cancer.

**Table 1:** Literature review of asymptomatic patients.

Case	Age (yrs)	Histopathologi caldiagnosis	ER	PR	Hormone therapy (medicine)	When metastasis was discovered	How to find metastasis	Major treatment since diagnosis of AUM
Charvolin et al,^[9]^ 2002	51	IDC	NA	NA	NO	During the follow-up	Pelvic ultrasound + tumor marker	Surgery + chemotherapy
Acikalin et al,^[10]^ 2005	58	IDC	+	+	YES (tamoxifen)	During the follow-up	Endometrial curettage	Surgery
Perisic et al,^[11]^ 2007	65	ILC	+	+	YES (tamoxifen)	During the follow-up	Gynecological examination + biopsy	NA
Bogliolo et al,^[12]^ 2010	78	ILC	+	+	NO	Before the diagnosis of breast cancer	MRI	Chemotherapy + radiotherapy
İşçi et al,^[13]^ 2011	47	ILC	+	-	YES (letrozole)	During the follow-up	CT	Surgery + chemotherapy
Dirican et al,^[14]^ 2012	51	IDC	-	+	YES (tamoxifen)	During the follow-up	CT + tumor marker	Surgery + chemotherapy
van et al,^[15]^ 2012	70	IDC	NA	NA	YES (tamoxifen)	During the follow-up	CT	Surgery
Munoz-Iglesias et al,^[16]^ 2013	50	ILC	NA	NA	YES (NA)	During the follow-up	Tumor marker + PET/CT	Surgery
Proenca et al,^[17]^ 2016	58	IDC	+	+	YES (tamoxifen)	During the follow-up	Cervical cytology	Chemotherapy
	77	ILC	+	+	YES (anastrozole + tamoxifen)	During the follow-up	Gynecological examination + cervical cytology	Radiotherapy

ER = estrogen receptor, PR = progesterone receptor, AUM = asymptomatic uterine metastasis, IDC = invasive ductal carcinoma, NA = not available, ILC = invasive lobular carcinoma, MRI = magnetic resonance imaging, CT = computed tomography, PET/CT = positron emission tomography/computed tomography.

Recently, an increasing number of studies have shown that radiomics features correlate with the biological behavior of tumors to a large extent, confirming the feasibility of differentiating tumor tissues and helping to assess therapeutic effects and the prognosis of tumors.^[22]^ Compared with traditional imaging diagnosis methods, radiomics can objectively and noninvasively evaluate heterogeneity among lesions and organs and reflect relevant information of the organizational microenvironment.^[23]^ After quantizing the images with the proper number of gray levels, magnetic resonance imaging (MRI) texture features can be used to identify brain metastases from different primary cancers.^[24]^ Overall, further study and exploration are needed to determine whether radiomics can be used to identify tumors of the female reproductive system that cannot be distinguished by the naked eye.

**Figure 4.**
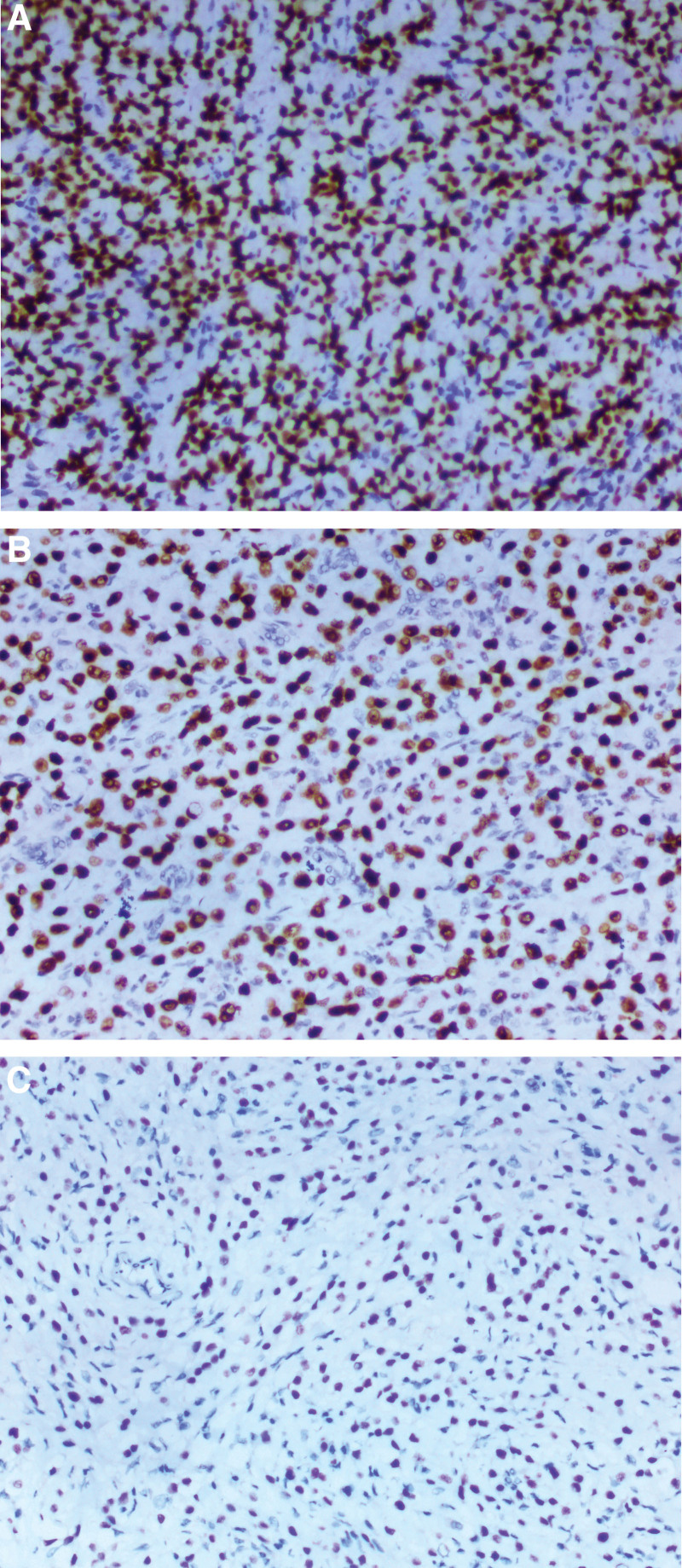
Immunostaining showing the neoplasm to be strongly and diffusely positive for GATA-3 in cervical curettage pathology (A), uterus postoperative pathology (B), and pathology of the left cervical lymph node (C). The immunoprofile supports the diagnosis of metastasis as primary breast carcinoma. (A–C, EnVision method, original magnification × 200).

Mazur et al reported that approximately 42% of metastatic cervical tumors were mistaken for primary cervical tumors.^[2]^ Pathological biopsy is usually the strictest criterion for distinguishing a primary uterine mass from a secondary mass of extragenital metastasis. If the imaging examination is highly suspicious but difficult to distinguish based on the imaging findings, accurate immunohistochemical examination is required. Among the many immunohistochemical indices, GATA-3 may be a reliable choice. GATA-3 is 1 of the 6 members of the zinc finger transcription factor family.^[25]^ It recognizes a specific nucleotide sequence in the promoter of target genes and has an important role in regulating breast morphogenesis and lumen cell differentiation. GATA-3 has an important role in tumorigenesis.^[26]^ Mazoujian et al reported that approximately 55% of breast cancers are positive for gross cystic disease fluid protein-15 (GCDFP-15) and that 90% of them are invasive lobular carcinomas.^[27]^ Haiyan Liu et al performed immunohistochemical evaluation of GATA-3 expression in 1110 cases of cancer and 310 normal tissues. GATA-3 was highly expressed in both breast cancer and urothelial carcinoma. The expression rates of IDC and ILC were 91% and 100%, respectively, but that of endometrial carcinoma was only 2%, and no expression in cervical adenocarcinoma or ovarian serous carcinoma was found. The rate of GATA-3 positivity shows little change in both primary and metastatic breast cancer.^[28]^ A study that compared the value of GATA-3, mammaglobin (MGB), and GCDFP-15 for pathological diagnosis of breast cancer reported a positive rate for GATA-3 (92.5%; 94.25%) that was significantly higher than that for MGB (42.11%; 29.17%) and GCDFP-15 (55.77%; 31.34%) in primary or metastatic breast cancer. GATA-3 is a sensitive marker for evaluating tumors of unknown origin, especially for malignant effusion.^[29]^ As the case we report was positive for GATA-3 by cervical curettage pathology, uterus postoperative pathology, and pathology of the left cervical lymph node, it can be confirmed that the tumors in the above sites all originated from the breast (Fig. 4).

GATA-3 = GATA binding protein 3.

According to the latest guidelines, the patient accepted paclitaxel liposome (175 mg/m²) + capecitabine (1000 mg/m²) chemotherapy for 6 cycles. Regarding the uterine metastasis, she received surgery and postoperative radiotherapy; the treatment was replaced with gemcitabine (1000 mg/m²) + cisplatin (75 mg/m²) for systemic chemotherapy after recurrence. However, the patient finally refused further treatment for personal reasons and died at home in August 2021.

## 4. Conclusion

Asymptomatic uterine metastases from breast cancer are rare. Our case focuses on the diagnosis and treatment of a patient with asymptomatic uterine metastasis from breast cancer, and summarizes the previous asymptomatic patients with metastases. During reexamination of breast cancer patients, clinicians must be alert to metastasis to gynecologic organs, especially in patients with hormone receptor positive and taking tamoxifen. Regular review of asymptomatic patients is highly warranted. How to predict and detect distant metastases in advance remains to be discussed in further research. If there is an abnormal sign in the uterus, diagnostic curettage or pathological biopsy can help in definitive diagnosis. Immunohistochemistry for GATA-3 may indicate that the tumor originated from the breast. At present, there is still no targeted treatment of uterine metastasis of breast cancer, and it is necessary to adopt TAH and BSO to prevent the residual tumor tissue from spreading to other organs in the abdominal cavity, and postoperative radiotherapy should be supplemented. Asymptomatic patients with breast cancer metastasizing to the uterus are very rare, and further research is needed for their diagnosis and treatment.

## Acknowledgments

We thank the patient, who agreed to the publication of her images and clinical information. Informed consent was obtained from the patient for publication of the case.

## Author contributions

Dechen Kong: Retrieved clinical data; wrote and edited the manuscript. Xiaotong Dong and Furong Hao: Captured biopsy images; assisted with figure development. Peiyan Qin and Zhengtao Zhang: conceived this article; retrieved clinical data. Daqing Sun and Yan Zhang: supervised the writing; assisted with editing the manuscript. Mingchen Wang: conceived, designed, and supervised this study.

**Conceptualization:** Peiyan Qin, Zhengtao Zhang.

**Data curation:** Dechen Kong, Peiyan Qin, Zhengtao Zhang.

**Investigation:** Dechen Kong.

**Supervision:** Daqing Sun, Yan Zhang, Mingchen Wang.

**Visualization:** Xiaotong Dong, Furong Hao.

**Writing – original draft:** Dechen Kong.
